# Association between Life’s Essential 8 and cognitive function: insights from NHANES 2011–2014

**DOI:** 10.3389/fnagi.2024.1386498

**Published:** 2024-04-04

**Authors:** Kangni Liang, Xiaoling Zhang

**Affiliations:** ^1^Zhejiang Chinese Medical University, Hangzhou, China; ^2^Department of Neurology, The Second Affiliated Hospital of Jiaxing University, Jiaxing, China

**Keywords:** cognitive function, Life’s Essential 8, NHANES, older adults, cardiovascular health

## Abstract

**Introduction:**

Life’s Essential 8 (LE8) is prompted by the American Heart Association (AHA) to assess cardiovascular health. The association between LE8 and cognitive function in America is unknown. Our study was to investigate the association of LE8 with cognitive function in general adults.

**Materials and methods:**

A total of 2,301 participants were enrolled in the National Health and Nutrition Examination Surveys (NHANES). LE8 scores (range 0–100) were obtained from measurements based on American Heart Association definitions, divided into health behavior and health factor scores. Cognitive function was assessed by three tests including the Consortium to Establish a Registry for Alzheimer’s Disease (CERAD), Animal fluency test (AFT), and Digit Symbol Substitution test (DSST). The multivariable linear regression analysis explored the associations between LE8 and cognitive function. Smooth curve fitting was explored using restricted cubic splines. The inflection point was determined by the two-piecewise linear regression.

**Results:**

In the multivariable linear regression model with full adjustment for confounding variables, AFT scores were 1.2 points higher in participants with LE8 scores >80 than in those with LE8 scores <50 (high LE8 score group: *β* = 1.20, 95% CI 0.37, 2.03), and 3.32 points higher in DSST (high LE8 score group: *β* = 3.32, 95% CI 1.24, 5.39). Although high LE8 scores show a Negative association with high CERAD, we found a significant association between higher LE8 scores and higher CERAD when LE8 scores were higher than 82.5 (*β* = 0.21 95%CI 0.04, 0.39, *p*-value = 0.0179).

**Conclusion:**

Our study highlighted a positive association between Life’s Essential 8 and cognitive function in older adults.

## 1 Introduction

Cognitive impairment is a pathological process in which there are abnormalities in the brain’s higher intellectual processing related to learning and memory as well as thinking and judgment, resulting in learning and memory impairments accompanied by changes such as aphasia or dysarthria or dyscognition or dysarthria (US Preventive Services Task Force et al., 2020). Cognitive impairment can occur in a variety of disorders such as Alzheimer’s disease, vascular cognitive impairment and Parkinson’s disease. Cognitive impairment can be categorized into several stages, from mild cognitive impairment (MCI) which is a transitional state between normal cognition and dementia to full-blown dementia (most commonly Alzheimer’s disease) (Gaugler et al., 2022). The literature describes that about 20% of patients with MCI will develop dementia over time (Qin et al., 2023). The number one cause of dementia is Alzheimer’s disease (AD) which is one of the costliest and a serious burden on society. Approximately 47 million people were living with dementia globally in 2015. By 2050 this number will increase to 131 million (Orgeta et al., 2019), with annual global losses due to dementia exceeding. The annual global cost of dementia is more than $800 billion, more than 85% of which involves family and social costs rather than healthcare costs (Lancet, 2019). In many countries, the age-specific incidence of dementia has already declined. The Commission on Dementia Prevention, Intervention, and Care has identified 12 potentially modifiable risk factors for dementia (Livingston et al., 2020): lower educational attainment, high blood pressure, hearing impairment, smoking, obesity, depression, physical inactivity, diabetes, low social contact, excessive alcohol consumption, traumatic brain injury (TBI), and air pollution.

In 2010, the American Heart Association (AHA) defined the concept of Life’s Simple 7 (LS7) (Lloyd-Jones et al., 2010), which is used to assess cardiovascular health and promote a shift from focusing solely on disease treatment to actively promoting and protecting health throughout the life course of populations and individuals. 2022 Life’s Essential 8 (LE8) provides a more comprehensive approach to assessing and improving an individual’s heart health (Lloyd-Jones et al., 2022; Shetty et al., 2023). LE8 recognizes the importance of sleep in preventing heart disease and other cardiovascular diseases and is more comprehensive and sensitive to inter-individual variations than LS7. LE8 incorporates diet, physical activity, nicotine exposure, sleep health, body mass index, lipids, blood glucose, and blood pressure. These 8 items are scored on a scale of 0–100, and the total LE8 score is calculated as the arithmetic mean of the 8 indicators, which we labeled quantifying cardiovascular health (CVH value). These 8 domains highly overlap with the 12 dementia risk factors.

There is previous evidence of an association between LS7 and cognitive impairment in the United States (González et al., 2018; Samieri et al., 2018; Guo et al., 2021). Since the concept of LE8 was introduced, there have been 2 papers in the United Kingdom demonstrating an association between LE8 and dementia (Petermann-Rocha et al., 2023; Wang et al., 2023), but the study populations in these papers were mainly based on European populations and lacked generalizability. Moreover, the possible association of LE8 with cognitive impairment in the United States remains unattended by many scholars. Therefore, to complement this information and add to the evidence of a possible link between LE8 and cognitive impairment, this study aimed to utilize the 2011–2014 National Health and Nutrition Examination Survey (NHANES) to investigate the association between LE8 and cognitive function in the US population. We hypothesized that the level of LE8 scores would be positively associated with scores of cognitive functions and negatively associated with the likelihood of cognitive impairment.

## 2 Materials and research methods

### 2.1 Date sources

Cross-sectional data were obtained from NHANES, a national cross-sectional study designed to assess the health and nutritional status of the general U.S. population administered by the National Center for Health Statistics (NCHS) through interviews and examinations. Due to the stratified multistage probability sampling methodology used in the NHANES study design, the inclusion sample was highly representative. All NHANES data used in our analysis are available at https://www.cdc.gov/nchs/nhanes/.

### 2.2 Study population

**Figure 1:**
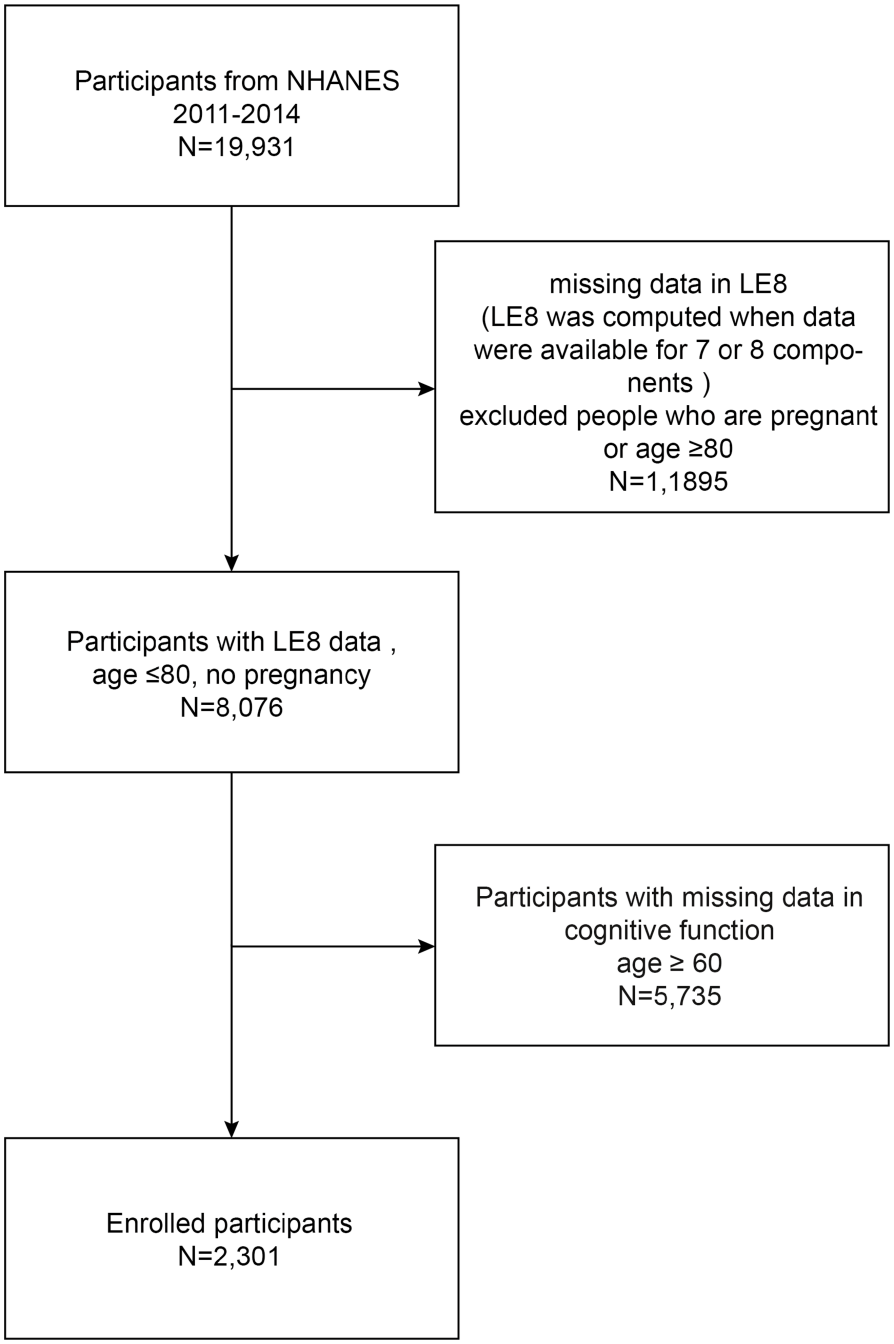
Participants included in the study.

As shown in Figure 1, our study was based on data obtained from two 2-year NHANES surveys conducted between 2011 and 2014. Initially, there were 19,931 participants, of whom 11,895 were cleared because of missing data for LE8 value calculations, pregnancy, and age 80 years or older. Pregnant and 80+ year olds were excluded because their cardiovascular function differed significantly from that of others and the cardiovascular health (CVH) calculation error was large. After excluding participants with missing data on cognitive function (*n* = 5,735), the final analysis covered 2,301 participants (see flow chart).

### 2.3 Ethical considerations

The National Center for Health Statistics Research Ethics Review Board approved the implementation of NHANES involving human subjects, and all participants provided informed consent.

### 2.4 Independent variable: Life’s Essential 8

In recent years, the social determinants of health and the underlying environment of mental health have also been recognized as key factors in optimizing and protecting cardiovascular health. As a result of these studies, the American College of Cardiology recently published a revised conceptualization and improved instrument for assessing cardiovascular health, the Life Essentials 8 (LE8), which consists of 2 major domains: health behaviors, including dietary health, physical activity health, nicotine exposure, and sleep health; and health factors, including body mass index, lipids, blood glucose, and blood pressure, with a score ranging from 0 to 100 for each of the 8 indicators. See references for detailed evaluation criteria (Shetty et al., 2023).

The total LE8 score was calculated as the arithmetic mean of the 8 indicators, which we labeled as CVH values, and in this paper, we will primarily use CVH values to represent the LE8 scores. Researchers do not strictly group CVH, but we found that previous studies (Lloyd-Jones et al., 2022; Yi et al., 2023) have considered participants with CVH values of 80–100 to be in the high CVH group; participants with CVH scores of 50–79 to be in the medium CVH group; and participants with CVH scores of 0–49 to be in the low CVH group. To investigate the association between CVH and cognitive function scores, the above method of grouping was also used in this study (Lloyd-Jones et al., 2022).

In this study, the participants will be scored strictly according to the scoring criteria issued by the AHA, and the association between LE8 and cognitive function scores will be observed by using the CVH total score as the main observation, and the detailed algorithm is shown in the relevant literature.

### 2.5 Dependent variable: cognitive function

We used the Consortium to Establish a Registry for Alzheimer’s disease (CERAD), Animal fluency test (AFT), and Digit Symbol Substitution test (DSST) to assess cognitive performance. Cognitive function can be categorized into specific cognitive domains such as processing speed, attention, memory, language, visuospatial abilities, and executive functioning (Harada et al., 2013).

The CERAD test consists of three consecutive learning tests and one delayed recall test. The maximum score for each test is 10 points, and the final CERAD score is the sum of the three learning tests (CERAD-WL) and the recall test (CERAD-DR), which assesses both immediate and delayed learning, and can evaluate cognitive functions such as memory, language, and attention (Casagrande et al., 2021). The Animal Naming Test (AFT) assesses cognitive functions such as executive functioning and verbal fluency by asking participants to name as many animals as they can in 1 min, scoring one point for each animal name, on a scale of 1–40 (Bailey et al., 2020). The DSST uses a paper test with a key at the top that has 9 pairs of numbers and symbols on it. Subjects are asked to match the corresponding symbols from 133 boxes next to the numbers within 2 min. The total score is based on the number of correct pairs and assesses participants’ processing speed, sustained attention, and working memory on a scale of 1–100 (Clark et al., 2009).

It is important to note that although there are no recognized thresholds for the CERAD, AFT, and DSST to differentiate between cognitive impairments, it is generally accepted that the higher the score, the better the participant’s cognitive functioning in terms of memory, concentration, and executive functioning (Sutin et al., 2022; Li W. et al., 2023). And some prior researches (Bailey et al., 2020; Gong et al., 2021), cutoffs of <14 for AFT, <34 for DSST were used to distinguish potential cognitive impairment from healthy cognitive function and lack of cognitive impairment in the NHANES.

The three measurement used in this study, although cannot fully replace a diagnosis based on a clinical examination, can be utilized to observe the relationship between certain factors and cognitive functions (Lee et al., 2021).

### 2.6 Covariates

Our multivariable-adjusted model summarized potential covariates that could confound the association between LE8 and cognitive function. Due to the large number of projects in LE8, fewer covariates were adjusted in this study to prevent model overfitting (Tian et al., 2024). Covariates in our study included age, sex (male or female), race (Mexican American, other Hispanic, non-Hispanic white, non-Hispanic black, other race), educational attainment (high school and below, some college and above), and poverty ratio (PIR).

### 2.7 Statistical analysis

Continuous variables are presented as means and standard errors (SE) and categorical variables are presented as percentages. Weighted Student’s *t*-tests (for continuous variables) or weighted chi-square tests (for categorical variables) were used to assess differences within groups. Pearson correlation analyses were conducted to test the relationship between covariates and cognitive functioning.

Multivariate linear regression analyses were used to explore the independent relationship between CVH scores and cognitive functioning scores in three different models. Model 1 was an unadjusted model with no adjustment for covariates. Model 2 was a crude adjusted model, adjusted for gender, age, and ethnicity. Model 3 was a fully adjusted model, adjusted for gender, age, race, education level, and income. As mentioned above, the CVH total score was further analyzed for sensitivity in this study in 3 separate groups. In addition, an interaction test was added to analyze whether covariates were likely to influence the association of CVH scores with cognitive functioning. Values of *p* < 0.05 were considered statistically significant.

All analyses were performed using Empower software^1^ (X&Y Solutions, Inc., Boston MA) and R version 4.1.2 (The R Foundation).^2^

## 3 Results

### 3.1 Baseline characteristics

The weighted baseline characteristics of the included individuals are shown in Table 1. A total of 2,301 participants were included in our analysis, with a mean age of 69.40 ± 6.75 years, 49.59% were male and 50.41% were female. The overall mean CVH value was 60.33 ± 13.99, the mean CERAD value was 25.12 ± 6.45, the mean AFT value was 16.87 ± 5.49, and the mean DSST value was 46.90 ± 17.03, and participants with a high CVH tended to have higher cognitive scores and better cognitive functioning compared to those with a low CVH (*p* < 0.05). In the case of AFT, for example, CVH <50: 15.78 ± 5.39; CVH ≥50, <80: 16.97 ± 5.37; and CVH ≥80: 18.99 ± 5.95. As shown previously, it has been argued that AFT <14 is the one cutoff for cognitive impairment, so that a portion of the population with AFT values in the low CVH group should be within the group of cognitive impairment. We found that participants in the high CVH group were more likely to be Non-Hispanic White, were often postgraduate and above, and tended to have higher incomes compared to the low CVH group. These covariates were summarized in our further multivariate-adjusted regression models with subgroup analyses as shown in Tables 2–4.

**Table 1:** Baseline characteristics of study population according to CVH scores.

CVH score	<50	>=50, <80	>=80	*p*-value
	*N*=549	*N*=1541	*N*=211	
Age, y	68.25±6.30	69.76±6.81	69.69±7.06	<0.001
Gender, %				0.541
Male	261(47.54%)	773(50.16%)	107(50.71%)	
Female	288(52.46%)	768(49.84%)	104(49.29%)	
Race,%				<0.001
Mexican American	55(10.02%)	128 (8.31%)	13 (6.16%)	
Other Hispanic	62 (11.29%)	153 (9.93%)	7 (3.32%)	
Non-Hispanic White	227(41.35%)	816(52.95%)	133(63.03%)	
Non-Hispanic Black	182(33.15%)	316(20.51%)	20(9.48%)	
Other Race - Including Multi-Racial	23(4.19%)	128(8.31%)	38(18.01%)	
Education,%				<0.001
High school and below	341 (62.11%)	708 (45.94%)	31 (14.69%)	
Some college and above	208 (37.89%)	833 (54.06%)	180 (85.31%)	
PIR	2.10 ± 1.48	2.71 ± 1.57	3.72 ± 1.47	<0.001
Diet score	30.09±27.39	55.14±29.62	81.87±21.25	<0.001
Physical activity score	6.87±22.32	42.21±46.07	92.75±19.86	<0.001
Nicotine exposure score	56.62±37.06	78.59±27.98	88.86±18.33	<0.001
Sleep health score	69.64±29.56	85.32±22.27	93.74±14.53	<0.001
Body mass index score	41.36±32.48	61.89±30.60	88.98±17.29	<0.001
Blood lipids score	49.25±30.45	64.57±27.31	75.55±24.86	<0.001
Blood glucose score	52.50±26.63	71.71±25.96	90.05±17.50	<0.001
Blood pressure score	30.45±26.10	48.13±30.86	68.34±28.48	<0.001
CERAD test	24.50±6.40	25.08±6.37	27.03±6.76	<0.001
Animal fluency test	15.78±5.39	16.97±5.37	18.99±5.95	<0.001
DSST	42.09±16.74	47.33±16.81	56.22±15.04	<0.001

**Table 2:** Subgroup analysis for the association between CVH Score and CERAD test.

	CVH Score	*p*-value
Age		**0.0162**
<70	0.02 (0.00, 0.05) **0.0440**	
>70	−0.02 (−0.05, 0.01) 0.1852	
Gender		0.4207
Male	0.00 (−0.02, 0.03) 0.7237	
Female	0.02 (−0.01, 0.04) 0.1424	
Race		0.8364
Mexican American	0.01 (−0.05, 0.07) 0.8030	
Other Hispanic	0.04 (−0.02, 0.10) 0.1774	
Non-Hispanic White	0.01 (−0.01, 0.04) 0.3490	
Non-Hispanic Black	0.01 (−0.03, 0.05) 0.6431	
Other Race - Including Multi-Racial	−0.01 (−0.07, 0.05) 0.7735	
Education		0.8379
High school and below	0.01 (−0.02, 0.04) 0.4900	
Some college and above	0.01 (−0.01, 0.04) 0.2800	
PIR		0.6844
Q1	0.01 (−0.02, 0.04) 0.6697	
Q2	0.02 (−0.01, 0.05) 0.1469	
Q3	0.01 (−0.02, 0.04) 0.7017	

Bold value indicates *p*-value <0.05.

**Table 3:** Subgroup analysis for the association between CVH Score and Animal fluency test.

	CVH Score	*p*-value
Age		**0.0227**
<70	0.04 (0.02, 0.06) **0.0004**	
>70	0.00 (−0.02, 0.02) 0.8964	
Gender		0.7682
Male	0.02 (0.00, 0.04) **0.0347**	
Female	0.03 (0.01, 0.05) **0.0101**	
Race		**0.0017**
Mexican American	0.03 (−0.02, 0.08) 0.2332	
Other Hispanic	0.05 (0.00, 0.10) **0.0401**	
Non-Hispanic White	0.04 (0.02, 0.06) **0.0004**	
Non-Hispanic Black	0.02 (−0.01, 0.05) 0.2473	
Other Race - Including Multi-Racial	−0.07 (−0.12, −0.02) **0.0053**	
Education		0.5149
High school and below	0.02 (−0.00, 0.04) 0.0954	
Some college and above	0.03 (0.01, 0.05) **0.0045**	
PIR		**0.0353**
Q1	0.02 (−0.01, 0.04) 0.2186	
Q2	0.01 (−0.02, 0.03) 0.6432	
Q3	0.05 (0.03, 0.08) **<0.0001**	

Bold value indicates *p*-value <0.05.

**Table 4:** Subgroup analysis for the association between CVH Score and DSST.

	CVH Score	*p*-value
Age		0.2892
<70	0.10 (0.05, 0.15) **0.0002**	
>70	0.06 (−0.00, 0.12) 0.0614	
Gender		0.3161
Male	0.11 (0.06, 0.17) **<0.0001**	
Female	0.08 (0.03, 0.13) **0.0032**	
Race		0.5688
Mexican American	0.09 (−0.04, 0.22) 0.1601	
Other Hispanic	0.11 (−0.02, 0.24) 0.0876	
Non-Hispanic White	0.12 (0.07, 0.17) **<0.0001**	
Non-Hispanic Black	0.04 (−0.04, 0.12) 0.3237	
Other Race - Including Multi-Racial	0.09 (−0.04, 0.22) 0.1740	
Education		0.6989
High school and below	0.09 (0.03, 0.14) **0.0029**	
Some college and above	0.10 (0.05, 0.15) **<0.0001**	
PIR		0.7828
Q1	0.08 (0.01, 0.15) **0.0184**	
Q2	0.11 (0.05, 0.18) **0.0008**	
Q3	0.09 (0.03, 0.16) **0.0041**	

Bold value indicates *p*-value <0.05.

### 3.2 The higher the CVH score, the higher the cognitive function score

Table 5 shows the associations between the total CVH scores and the items and cognitive function scores. It is evident that in the unadjusted model and the partial whole model, participants with higher CVH also had higher CERAD, AFT, and DSST scores. After full adjustment, we observed that: in the CERAD test, the β-values of the medium and high CVH groups were 0.27 and 0.87, respectively, compared to the group with a CVH value of <50; medium CVH group: *β* = 0.27, 95% CI −0.30, 0.85; high CVH group: *β* = 0.87, 95% CI −0.11, 1.85; and in the AFT, *β*-values of 0.58 and 1.20, medium CVH group: *β* = 0.58, 95%CI 0.09, 1.07; high CVH group: *β* = 1.20, 95%CI 0.37, 2.03; in DSST, β values were 1.87 and 3.32, medium CVH group: *β* = 1.87, 95%CI 0.65, 3.09; high CVH group: *β* = 3.32, the 95%CI 1.24, 5.39.

**Table 5:** Association between LE8 and cognitive performance.

	**Model1**	**Model2**	**Model3**
**CERAD test**			
CVH scores			
<50	0	0	0
>=50, <80	0.58 (−0.04, 1.21) 0.0669	0.86 (0.28, 1.45) 0.0040	0.27 (−0.30, 0.85) 0.3555
>=80	2.53 (1.51, 3.55) <0.0001	2.39 (1.43, 3.35) <0.0001	0.87 (−0.11, 1.85) 0.0815
Diet score	0.01 (−0.00, 0.01) 0.1306	0.01 (0.00, 0.02) 0.0126	0.00 (−0.01, 0.01) 0.7381
Physical activity score	0.01 (0.01, 0.02) <0.0001	0.01 (0.01, 0.02) <0.0001	0.00 (−0.00, 0.01) 0.1170
Nicotine exposure score	0.01 (−0.00, 0.02) 0.0544	0.01 (0.00, 0.02) 0.0453	−0.00 (−0.01, 0.01) 0.9965
Sleep health score	0.01 (0.00, 0.02) 0.0226	0.01 (0.00, 0.02) 0.0118	0.01 (-0.00, 0.02) 0.1804
Body mass index score	−0.01 (−0.02, −0.00) 0.0398	−0.00 (−0.01, 0.01) 0.5544	−0.00 (-0.01, 0.00) 0.2246
Blood lipids score	−0.01 (−0.02, −0.00) 0.0026	−0.00 (−0.01, 0.01) 0.9412	−0.00 (−0.01, 0.01) 0.5478
Blood glucose score	0.02 (0.01, 0.03) 0.0002	0.01 (0.00, 0.02) 0.0091	0.00 (−0.00, 0.01) 0.3463
Blood pressure score	0.02 (0.01, 0.03) <0.0001	0.01 (0.00, 0.02) 0.0215	0.01 (−0.00, 0.01) 0.1218
**Animal fluency test**			
CVH scores			
<50	0	0	0
>=50, <80	1.19 (0.67, 1.72) <0.0001	1.14 (0.64, 1.64) <0.0001	0.58 (0.09, 1.07) **0.0201**
>=80	3.21 (2.35, 4.07) <0.0001	2.80 (1.98, 3.62) <0.0001	1.20 (0.37, 2.03) **0.0045**
Diet score	0.01 (0.00, 0.02) 0.0053	0.02 (0.01, 0.02) <0.0001	0.01 (−0.00, 0.01) 0.0577
Physical activity score	0.02 (0.02, 0.03) <0.0001	0.02 (0.01, 0.02) <0.0001	0.01 (0.01, 0.02) **<0.0001**
Nicotine exposure score	0.01 (−0.00, 0.01) 0.1079	0.01 (0.00, 0.02) 0.0028	0.00 (−0.01, 0.01) 0.6376
Sleep health score	0.02 (0.01, 0.03) 0.0001	0.01 (0.00, 0.02) 0.0373	0.00 (−0.01, 0.01) 0.4450
Body mass index score	−0.00 (−0.01, 0.00) 0.1637	−0.00 (−0.01, 0.00) 0.5956	−0.00 (−0.01, 0.00) 0.1743
Blood lipids score	−0.01 (−0.02, −0.01) 0.0008	−0.01 (−0.01, 0.00) 0.0959	−0.01 (−0.02, −0.00) **0.0122**
Blood glucose score	0.02 (0.01, 0.03) <0.0001	0.01 (0.00, 0.02) 0.0014	0.00 (−0.00, 0.01) 0.2130
Blood pressure score	0.03 (0.02, 0.03) <0.0001	0.01 (0.01, 0.02) 0.0005	0.01 (0.00, 0.02) **0.0091**
**DSST**			
CVH scores			
<50	0	0	0
>=50, <80	5.24 (3.62, 6.86) <0.0001	4.87 (3.48, 6.26) <0.0001	1.87 (0.65, 3.09) **0.0027**
>=80	14.13 (11.48, 16.77) <0.0001	11.02 (8.73,13.30) <0.0001	3.32 (1.24, 5.39) **0.0017**
Diet score	0.05 (0.03, 0.07) <0.0001	0.06 (0.04, 0.08) <0.0001	0.01 (−0.00, 0.03) 0.0713
Physical activity score	0.09 (0.07, 0.10) <0.0001	0.07 (0.06, 0.08) <0.0001	0.04 (0.02, 0.05) **<0.0001**
Nicotine exposure score	0.06 (0.04, 0.09) <0.0001	0.06 (0.04, 0.08) <0.0001	0.02 (0.01, 0.04) **0.0104**
Sleep health score	0.06 (0.03, 0.09) <0.0001	0.04 (0.01, 0.06) 0.0035	0.00 (−0.02, 0.02) 0.7195
Body mass index score	0.00 (-0.02, 0.02) 0.8241	0.00 (−0.01, 0.02) 0.6014	−0.01 (−0.02, 0.01) 0.4029
Blood lipids score	−0.05 (−0.07, −0.02) 0.0002	−0.01 (−0.03, 0.01) 0.5633	−0.02 (−0.04, −0.00) **0.0431**
Blood glucose score	0.11 (0.09, 0.14) <0.0001	0.07 (0.05, 0.09) <0.0001	0.04 (0.02, 0.05) **0.0002**
Blood pressure score	0.08 (0.06, 0.10) <0.0001	0.03 (0.01, 0.05) 0.0007	0.02 (0.00, 0.03) **0.0281**

In sensitivity analysis, CVH Score was converted from continuous variable to categorical variable. It would be divided into three groups, including <50, ≥50 <80 and ≥80.

Diet score, physical activity score, nicotine exposure score, sleep health score, body mass index score, blood lipids score, blood glucose score and blood pressure score were converted from categorical variables to continuous variables.

Model 1: no covariates were adjusted.

Model 2: adjusted for sex, age and race.

Model 3: adjusted for sex, age, race, education level, PIR.

95% CI 95% confidence interval.

Bold value indicates *p*-value <0.05.

Although CVH scores and CERAD scores showed significant correlations in the unadjusted and partially adjusted models, such associations were not significant in the fully adjusted model, suggesting that there may be a link between positive correlations of CVH scores and CERAD scores, but that this link is not robust. However, in the remaining 2 tests, we observed relatively stable positive associations, and we observed that in the fully adjusted model, participants in the high CVH group had AFT scores and DSST scores that were 1.2 and 3.32 points higher than those in the low CVH group. There was a significant difference in the fact that those with greater cardiovascular fitness had certain aspects of cognitive functioning such as memory, executive functioning, and concentration compared with those with poorer cardiovascular health.

Finally, as a categorical item for LE8, we found that a healthy diet, higher intensity exercise, and good lipid and blood pressure control were significantly associated with better AFT and DSST scores, in addition to better DSST scores, which may also be associated with lower nicotine exposure and better glycemic control.

### 3.3 Smoothed curve and threshold effect analysis

**Figure 2:**
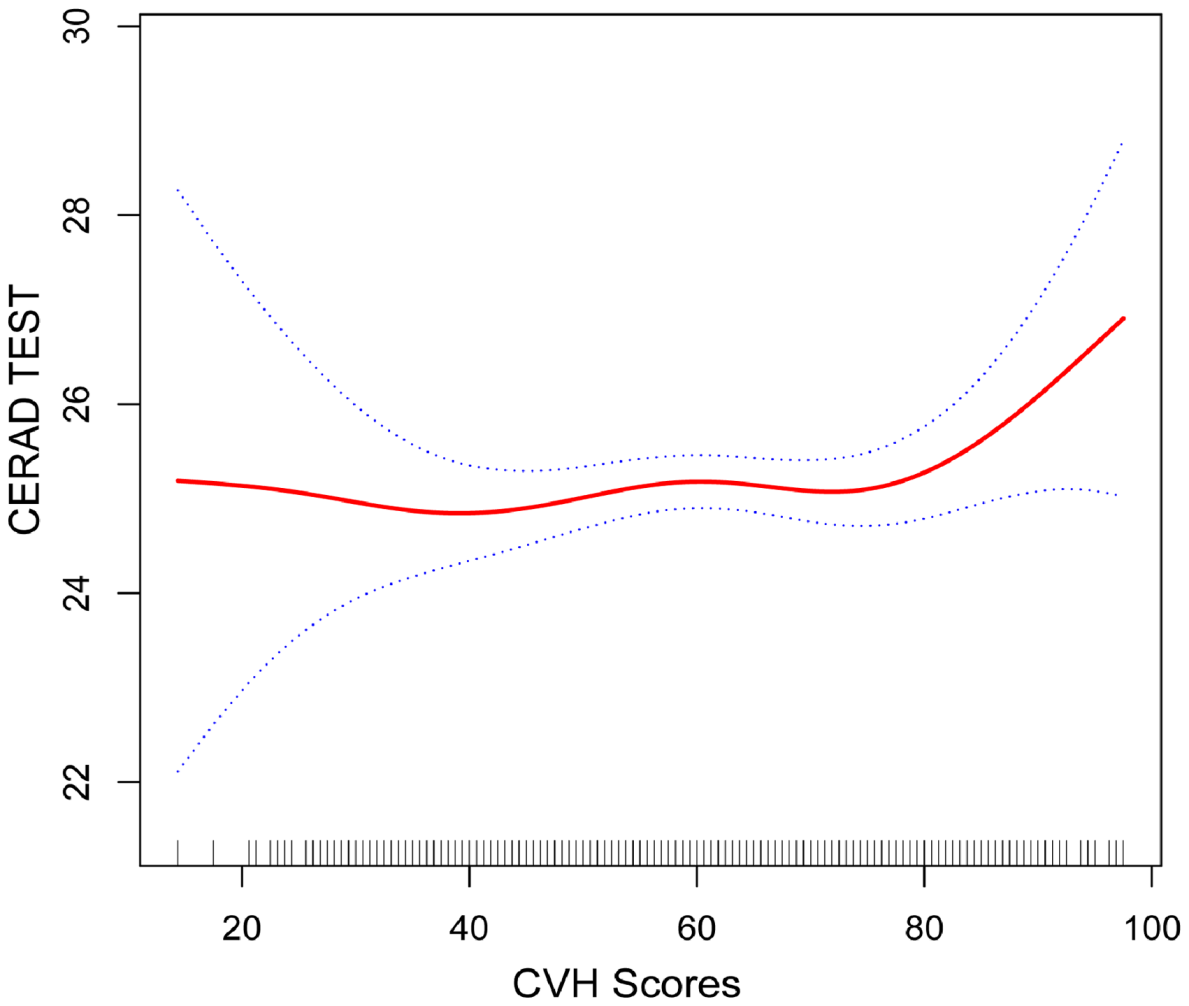
Smooth curve fitting detected a nonlinear positive relationship between CVH score and CERAD test was detected by the generalized additive model.

**Figure 3:**
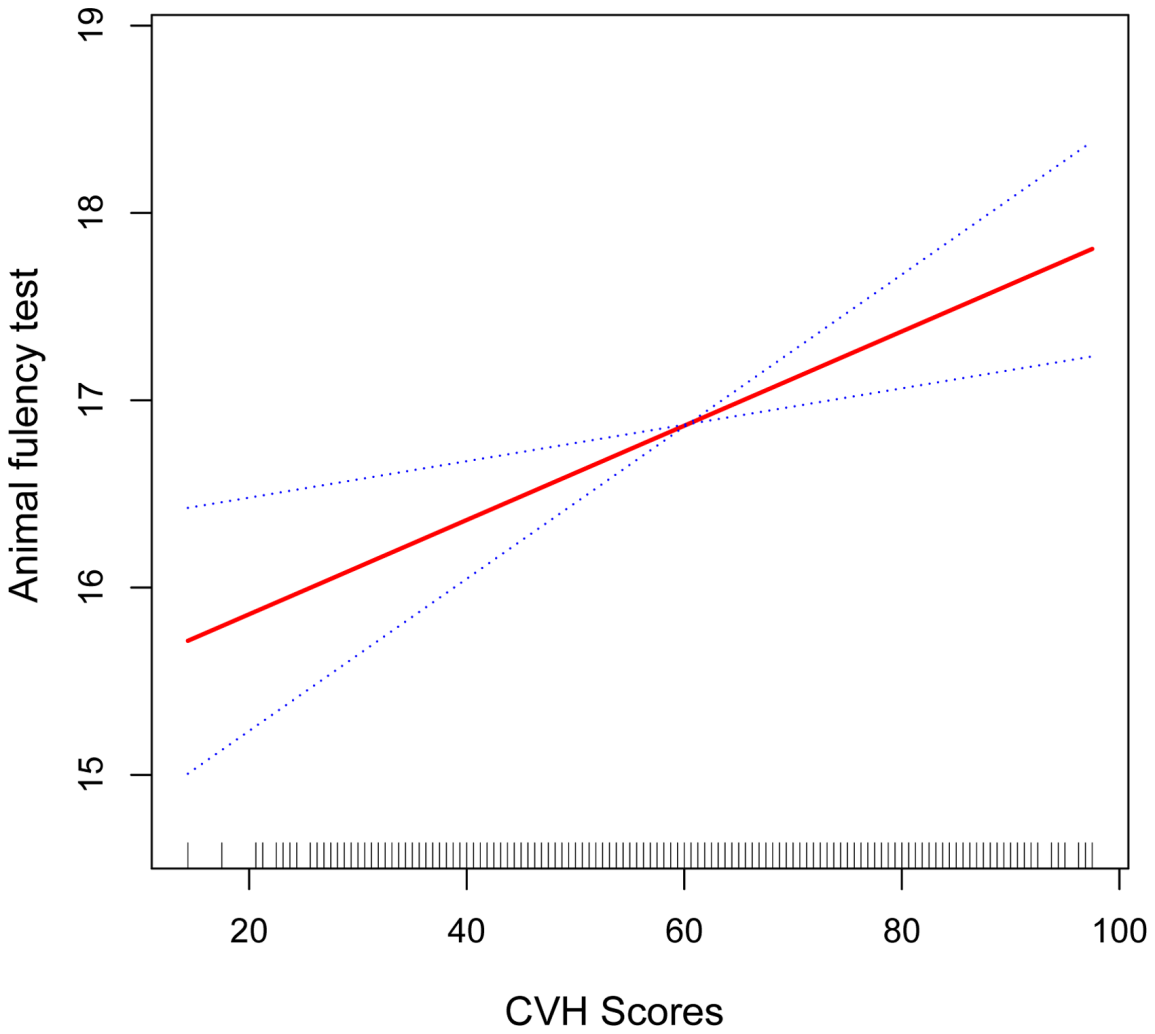
Smooth curve fitting detected a nonlinear positive relationship between CVH score and animal fluency test was detected by the generalized additive model.

**Figure 4:**
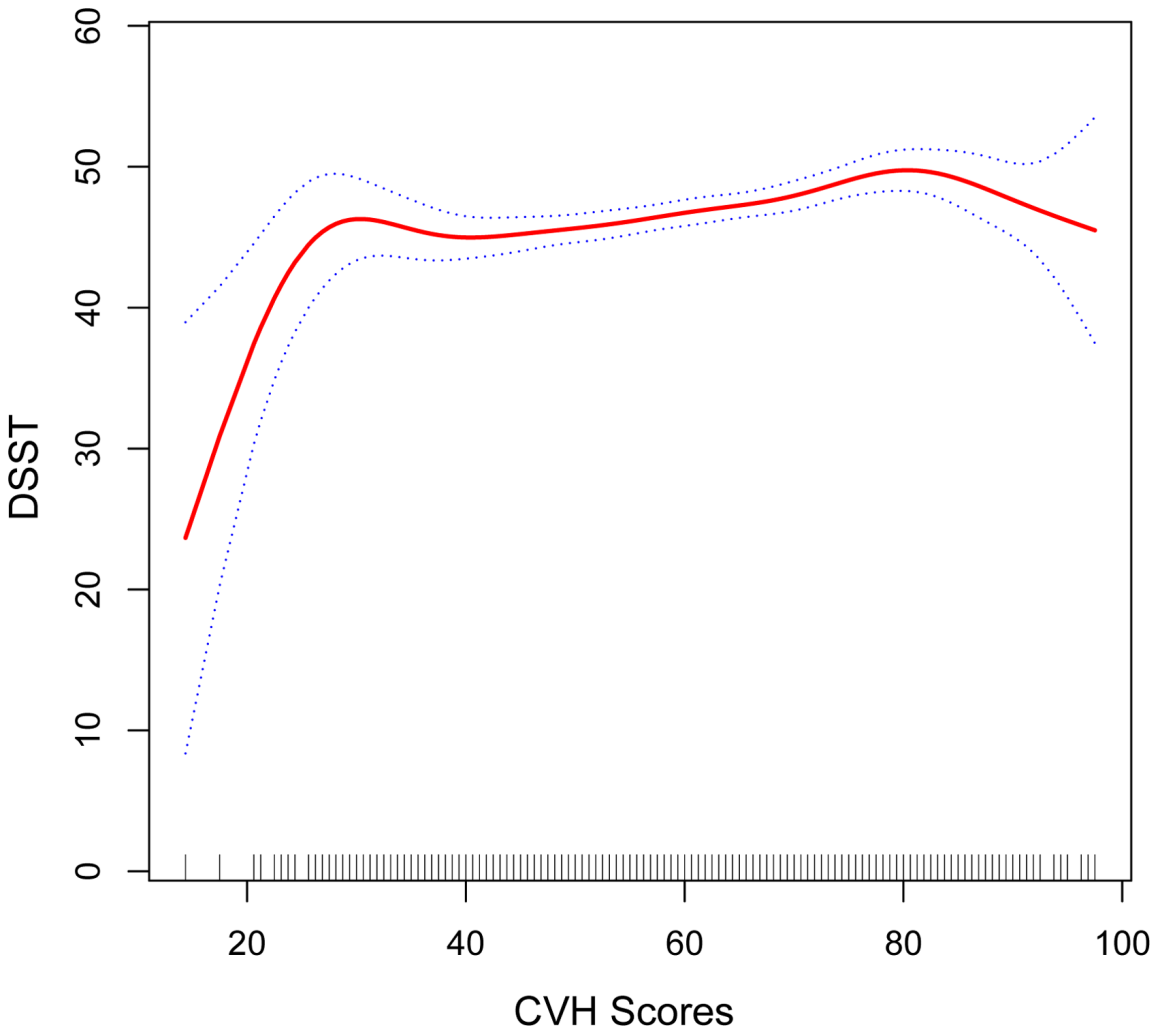
Smooth curve fitting detected a nonlinear positive relationship between CVH score and DSST was detected by the generalized additive model.

As shown in Figures 2–4, in both CERAD and DSST (Figures 2, 4), the smoothed curve fitting demonstrated a nonlinear relationship between CVH values and scale scores. We further calculated the fold point for the CVH and CERAD association to be 82.50. On the left side of the fold point, a negative correlation between CVH scores and CERAD scores was detected, which was not statistically significant (*β* = 0.00, 95% CI −0.02–0.02; *p* = 0.7642), and a positive correlation was detected on the right side of the fold point, which was statistically significant (*β* = 0.21, 95% CI 0.04–0.39; *p* = 0.0179), with a log-likelihood ratio test *p*-value of 0.024. This suggests that the rate of increase in CERAD scores is more strongly associated with the rate of increase in CVH scores when the CVH value is greater than 82.2, for every 1-point increase in the CVH value CERAD scale scores will increase by 0.21 points.

The fold point for the CVH and DSST association was 81.88, and a positive correlation between CVH scores and AFT scores was detected on the left side of the fold point, which was statistically significant (*β* = 0.11, 95% CI 0.07, 0.15; *p* < 0.0001), and a negative correlation was detected on the right side of the fold point, but it was not statistically significant (*β* = −0.22, 95% CI −0.56, 0.13; *p* = 0.2166), with a *p*-value of 0.072 for the log-likelihood ratio test.

Furthermore, we found that the smoothed curve fit in the AFT showed a linear relationship and that high scores on the CVH may be positively associated with high scores on the AFT scale (Figure 3).

### 3.4 Subgroup analyses

Subgroup analyses were conducted to further assess the robustness of the association between CVH assessment and cognitive function scores. In addition, because age, education, and poverty ratio are influential factors for cognitive impairment, they were tested for interaction in this study (Tables 2–4). To facilitate the test, to analyze the effect of age, we divided the population into 2 groups, using 70 years as the cut-off point; to analyze the effect of income, we triangulated the PIR as Q1, Q2, and Q3, respectively, with Q1 being the lowest income group.

After full adjustment, we detected a significant interaction of the age factor in CERAD and AFT (P for interaction 0.023; 0.016), with a greater effect value for CVH values when age < 70 years (*β* 0.02, 95% CI 0.00, 0.05, *p*-value 0.0440; β 0.04, 95% CI 0.02, 0.06, *p*-value 0.0004), i.e., the association between cognitive function and cardiovascular health is likely to be more significant when age is <70 years. In AFT, we also observed an interaction between race and income (P for interaction 0.0227; P for interaction 0.0017, respectively) In Other Hispanic, Non-Hispanic White, and Other Race the CVH values and the AFT were more significantly associated, and the association between the two was more significant among those with higher incomes.

In DSST, although no significant interaction was detected for age, we could still observe a significant association between CVH values and DSST scores when age was <70 years (β 0.10, 95%CI 0.05, 0.15, *p*-value 0.0002). Covariates such as age, gender, and race did not interact with the results of DSST scores, suggesting that the association between DSST scores and CVH values can remain stable across groups.

## 4 Discussion

This analysis used information from two NHANES cycles (2011–2012, 2013–2014) to examine the link between CVH levels and cognitive functioning. In this study, participants with high CVH values had AFT scores that were 1.2 points higher and DSST scores that were 3.32 points higher than those with low CVH values. So, this study presents a trend that participants with higher CVH values may have greater attention, memory, and executive functioning. Those under 70 years old showed a stronger association between CVH and cognitive function. This may suggest that early treatment and improvement of cardiovascular health may confer a greater benefit on cognitive performance.

A previous cohort study (Samieri et al., 2018) found that cardiovascular function scores were linked to cognitive performance and dementia risk. The study included 6,626 participants, and 745 dementia cases were adjudicated using the LS7 as an indicator of cardiovascular health assessment. It was found that the prevalence of dementia decreased with the number of cardiovascular health indicators that reached the optimal level after an average of 8.5 follow-up. Furthermore, Sabia et al. (2019) used the LS7 to measure cardiovascular health in 347 dementia cases over a 24-year period. They discovered that there was a negative correlation between cardiovascular scores and the likelihood of developing dementia, with higher cardiovascular health scores linked to a lower risk of dementia (hazard ratio of 0.89 for each 1-point increase in cardiovascular health scores). It has also been discovered that maintaining the LS7 at a desirable level can lower the risk of cerebral amyloid angiopathy. Researchers (López-Bueno et al., 2023; Yi et al., 2023) looked into the relationship between LE8 and cardiovascular mortality after learning about the AHA’s newer method of evaluating cardiovascular health. They discovered a significant inverse correlation between LE8 scores and the mortality from cardiovascular disease and all causes combined, demonstrating the validity of LE8 as a reliable indicator of cardiovascular health dependability. Thus, it is possible to employ the LE8 in study as a cardiovascular health indicator. The quartiles of LE8 scores were analyzed in a prospective study (Petermann-Rocha et al., 2023) involving 259,718 participants with a mean follow-up of 10.6 years. The results showed that individuals with the lowest LE8 scores had a 1.5 times higher risk of developing dementia than those with the highest scores, and that an increase of 10 points in the lowest quartile of scores would prevent 6.8% of cases of dementia caused by all causes. Nevertheless, this study was based on UK Biobank, which is an independent research organization that provides research on cardiovascular disease and cardiometabolic disease in the United Kingdom and the United States of America. The results, however, cannot be broadly applied because the study was based on UK Biobank and the subjects were primarily European. To strengthen the evidence supporting the relationship between LE8 scores and cognitive performance, research from other nations and ethnic groups is necessary. Our results are consistent with the generally accepted literature, which suggests that greater cardiovascular health scores are likely to relate to better cognitive function scores, so maintaining cardiovascular health is hoped to become a means to prevent cognitive impairments.

Our study adds the evidence that the population burden of cognitive impairment may be reduced by following the LE8 recommendations and cognitive gains are more pronounced for those younger than 70 years old. The latter demonstrates the importance of early prevention, starting as early as possible to emphasize the impact of cardiovascular health on cognitive impairment. Therefore early attention to the link between the two in the public health sector may help to improve people’s cognitive functioning and reduce the severe social burden caused by cognitive impairment (Petermann-Rocha et al., 2023). In addition, as many clinical trials as possible should be conducted to validate the link between LE8 and cognitive function, e.g., finding younger participants.

On multivariable linear regression analysis, we found that higher intensity exercise, healthy diet, and good lipid and blood pressure control were significantly associated with better AFT and DSST scores as categorical variables in LE8, which is consistent with previous studies. Previous studies (Angevaren et al., 2008) have shown that better physical activity improves cardiorespiratory fitness as well as cognitive function, and older adults with better balance tend to have less cognitive impairment (Meunier et al., 2021). Regarding diet, although a recent study (Barnes et al., 2023) showed no significant difference between participants with a family history of dementia and no cognitive impairment on the MIND diet and a mildly calorie-restricted control diet, there are studies (Millman et al., 2021; Roschel et al., 2021) that suggest that certain foods and nutrients, such as primary olive oil and creatine, have beneficial effects on cognitive function. Regarding lipids and blood pressure, a Chinese study (Lee et al., 2021) that included 2,215 participants showed that DSST scores increased with an increase in HDL (β coefficient: 0.036; *p* = 0.018), while a study by Liu et al. (2022) found a positive correlation between lipids and cognitive function in people with schizophrenia. Mahinrad et al. (2020) showed a positive correlation between blood lipids and cognitive function in people with schizophrenia, ranging from young to exposure to higher blood pressure levels during middle age was associated with poorer gait and cognitive performance during middle age.

When conducting subgroup analyses, we found that the health benefits of improved cardiovascular health were more pronounced when participants were < 70 years of age. This may be because age is an independent risk factor for dementia, with the risk of dementia progressively increasing as age rises. In addition, we found more significant associations between CVH values and AFT in Other Hispanic, Non-Hispanic White, Other Race, and higher income populations. Previous studies (González et al., 2018) examining the effect of midlife CVH values on cognitive function have found that the benefits of midlife CVH on cognitive performance were stronger in Whites than in Blacks, specifically, each unit increase in LS7 index had 1.8 times the effect on overall cognition in midlife compared with Whites, respectively. Poverty is an independent risk factor for dementia (Livingston et al., 2020), and one study (Lai et al., 2022) based on the UKB biobank found a higher risk of dementia among participants with low education, low household income, and unemployment relative to those with high education, high household income, and employment.

Many studies (Samieri et al., 2018; Sabia et al., 2019; John et al., 2021; Wu et al., 2021; Petermann-Rocha et al., 2023; Yi et al., 2023) have tried to demonstrat that a healthy cardiovascular system tends to result in better cognitive function. Recently, hypertension has been recognized the pathogenic factor both in cognitive impairment on vascular bases and in Alzheimer disease (AD) (Iadecola et al., 2019; Pacholko and Iadecola, 2024). There are some evidence (Wang et al., 2017) suggests that cardiovascular disease may underlie the pathophysiology of dementia. For example, cardiovascular disease is often accompanied by systemic inflammation and oxidative stress (Van Der Pol et al., 2019; Libby et al., 2021), and to date there is some evidence in the literature that inflammation and oxidative stress may be important mechanisms in the pathogenesis of dementia, particularly Alzheimer’s disease (Voet et al., 2019; Li H. et al., 2023). Besides, cardiovascular risk factors may affect cognitive function by increasing dementia-related neuroimaging markers (e.g., hippocampal atrophy, cerebral small vessel disease, and brain atrophy) (Baradaran et al., 2020). Cerebral vascular disease is a common independent contributor to age-related cognitive impairment (Greenberg, 2022), e.g., small vessel disease promotes ß-amyloid production, interferes with ß-amyloid clearance, and exacerbates the downstream consequences of ß-amyloid deposition (Greenberg et al., 2020). In addition, cardiovascular disease and dementia share many risk factors such as hypertension, high cholesterol, diabetes, obesity, smoking and physical inactivity, which not only increase the risk of atherosclerosis, but may also lead to cognitive impairment (Livingston et al., 2020; Wu et al., 2021).

The present study has some strengths. A key strength is the utilization of a sample of older adults drawn from the NHANES database, which is a nationally representative dataset, so our findings provide further evidence of the association between LE8 and cognitive function scores. To further show the consistency and robustness of our results, we carried out a number of subgroup studies, threshold effects investigations, and smoothed curve fitting procedures.

There are several limitations to this study. The study’s sample size was limited because cognitive function tests were only given to participants in the NHANES population during a specific annual cycle (2011–2014). Additionally, the study did not include cognitive function data from 2019 because field score data were stopped midway through the COVID-19 outbreak, leaving some data missing. Second, the NHANES database’s validation of causality is weak, and it is based on data from the National Health Survey, which does not provide temporal continuity for cohort research. Third, residual and unmeasured confounders still have the potential to affect study results, even though the analysis considered potential confounding variables related to individual characteristics at baseline. For this reason, the current study has employed rigorous statistical techniques to reduce the impact of confounders as much as possible. This study may still be biased in the ways listed below. Selection bias that might have arisen during the hiring process is one potential source. For instance, in NHANES, there is no data on cognitive testing for investigators under 60 years of age, which led to we cannot explore the relationship between LE8 and cognitive function in participants under 60 years. Meanwhile one literature show that associations of LE8 and cognitive function were stronger in people younger than 60 (Petermann-Rocha et al., 2023). So, this study does not represent the entire American, it excluded people <60 (*n* = 5,735). Measurement bias is another potential source of bias. Many of the participants’ lifestyles, comorbidities, and sociodemographic characteristics were obtained by self-report or recall; as such, they might be biased by either source. Consequently, care must be used when extrapolating the study’s findings because they are based on a nationally representative sample of Americans and might not be entirely applicable to other racial and ethnic groups outside of the country.

## Data availability statement

The original contributions presented in the study are included in the article/supplementary material, further inquiries can be directed to the corresponding author.

## Ethics statement

The studies involving humans were approved by the National Center for Health Statistics Research Ethics Review Board. The studies were conducted in accordance with the local legislation and institutional requirements. The participants provided their written informed consent to participate in this study.

## Author contributions

KL: Writing – original draft. XZ: Writing – review & editing, Writing – original draft.

## Footnotes

^1^www.empowerstats.com

^2^http://www.R-project.org
